# Radio-Oxidation Ageing of XLPE Containing Different Additives and Filler: Principal Component Analyses of Gases Emission and Consumption

**DOI:** 10.3390/polym14183810

**Published:** 2022-09-12

**Authors:** Lea Hippolyte, Sandrine Amat, Nathalie Dupuy, Muriel Ferry

**Affiliations:** 1 Aix Marseille Université, Avignon Université, CNRS, IRD, IMBE, 13013 Marseille, France; 2 CEA, Service D’Etude Du Comportement des Radionucléides, Université Paris-Saclay, 91191 Gif-sur-Yvette, France

**Keywords:** crosslinked polyethylene, radio-oxidation, formulation effect, dose effect, dose rate effect, chemometrics analysis

## Abstract

In the context of lifetime extension of Nuclear Power Plants (NPPs), electric cable ageing has to be checked to evaluate their performance during normal operation. These electric cables are complex materials, with a conductor and insulating shield in the metal and insulating layer and sheath in the polymer; the most sensitive layer is commonly considered to be the insulating layer. The ageing mechanism upon irradiation under oxidative conditions has been evaluated using gas mass spectrometry and the first conclusions have been drawn. Nonetheless, the data obtained are very numerous and complex; thus, the objective of this new article regards these experimental results using mathematical tools. It allowed confirmation of all the results obtained on these materials, but using chemometrics, i.e., statistical/mathematical analyses, of the results. Using these powerful mathematical tools gives strength to the analyses realised and to the conclusions obtained.

## 1. Introduction

In the face of global warming and electricity demand, which is expected to continue to grow in the coming years because of the increasing quantity of electric vehicles, increased digitalisation, etc., eminent climatologists and economists see nuclear energy as the ideal alternative to fossil fuels. Notably, in its report dated 6 October 2018 [1], the IPCC (International Group of Climate Experts, which presents the global scientific consensus on climate issues including the trajectories for reducing greenhouse gas emissions) indicates that nuclear power is an essential energy to limit global warming. Nuclear energy makes a significant contribution to reducing emissions and must continue to grow to meet the Paris Agreement targets [2]. In the short term, nuclear innovations are expected to contribute significantly to emission reduction targets. The rapid construction of new nuclear energy is possible. To maintain maximum safety conditions, the various elements of the power plants must be constantly checked: this is carried out through the intermediary of control cables that are the basic elements of the communication systems of the nuclear power plants.

Among the polymers mostly used in a wide range of applications is polyethylene (PE). It is used as common packaging plastic, single-use bags in biopharmaceutical manufacturing worldwide, automobiles, but also as electric cables insulation, including power cables in nuclear power plants (NPPs). In nuclear power plants, electric cables are complex elements that must transmit signals for control and instrumentation cables, and energy for power cables. During normal operation, the electric cables are subjected to different environments depending on their positioning and use. They may be exposed to gamma radiation and/or high temperatures. These conditions can induce physical or chemical changes, impact the material, and limit its lifespan. The factors that can influence the degradation of polymers under irradiation are, non-exhaustively, their chemical composition, additives, and presence of oxygen in the environment [3,4,5,6,7,8]. 

The European Union (EU) project TeaM Cables aims to develop models and algorithms to be able to foresee the aging of crosslinked polyethylene insulators. The methodology is based on multi-scale studies of their degradation under different conditions: gamma irradiation and/or thermal aging [9]. Many experimental results have been obtained, from the radicals’ identification [10] to the electrical behaviour [11], going through modifications at the molecular level [12,13,14].

Facing the results of quantity and complexity, mathematical analytical tools are sometimes needed to evaluate the influence of the different experimental factors, as well as their interactions. The Analysis in Common Dimension (AComDim) method [15] has, for instance, already successfully been applied for the detection of significant factors to analytical datasets from a variety of samples, e.g., to study the influence of the vintage (year) of wines, the iodine/epoxy-paint interactions in Nuclear Power Plants [16], the influence of the cultivar and the maturity, but also the humidity, shape, and lignin content effect on starch lignin mixtures [17]. In contrast to the analysis of variance–principal component analysis (ANOVA–PCA) method [18], AComDim replaces the separate PCAs with a single analysis to evaluate the significance of the effects [19].

Part of these experimental results have been presented in a previous article [20]. However, due to different irradiation conditions (irradiator, temperature in the irradiation chamber, and so on), the objective of the present paper is to analyse the obtained data using AComDim, which gives quantitative information about the variability in the quality indices resulting from the effect of polymer type, dose, dose rate, and modality of re-irradiation. The objective of this article is to evaluate, using both powerful mathematical methods, the influence of different ageing parameters on the radiation chemical yields of gases (formation and consumption) as input data.

## 2. Materials and Methods

### 2.1. Materials

Synthesis of the crosslinked polyethylene (XLPE) has already been presented in a previous article [20]. The Linear Low-Density Polyethylene (LLDPE) chosen for this study has a density of 0.918 g.cm^−3^ and melting point of 120 °C, and contains a very small quantity of antioxidants, mainly BHT and Irganox 1076 [13]. Two antioxidants and one flame retardant were added to the XLPE during its process: the antioxidants were Irganox 1076 and Irganox PS802, and the flame retardant was aluminium trihydrate (ATH).

Samples were furnished by Nexans France under the form of tapes of about 500 µm thick. In this work, different formulations were evaluated:

- Mod1: XLPE “as received”;
- Mod2: XLPE + 1 phr of Irganox 1076;
- Mod3: XLPE + 1 phr of Irganox PS802;
- Mod4: XLPE + 1 phr of Irganox 1076 + 1 phr of Irganox PS802;
- Mod5: XLPE + 25 phr of ATH;
- Mod6: XLPE + 50 phr of ATH;
- Mod7: XLPE + 1 phr of Irganox 1076 + 1 phr of Irganox PS802 + 50 phr of ATH.

### 2.2. Irradiation Conditions

Depending on the required irradiation conditions, two facilities were used: Panoza and Roza. In both of them, air flows inside the irradiator chamber and the temperature were roughly constant. The formulated polymer tapes were placed at a given and known distance from the source to obtain the desired dose rate, while the dose was determined using alanine dosimeters—with the uncertainty on these kinds of dosimeters at about 6.5%.

In this experiment series, two dose rates were employed, and for each dose rate, three doses were achieved:

- Low dose rate corresponded to 8.5 Gy·h^−1^ (irradiations at 45 °C, using Panoza). The three doses achieved at this dose rate were equal to 25 kGy, 67 kGy, and 138 kGy.
- Medium dose rate corresponded to 78 Gy·h^−1^ (irradiations at 45 °C, using Panoza). The three doses achieved at this dose rate were equal to 67 kGy, 220 kGy, and 374 kGy.
- High dose rate corresponded to 400 Gy·h^−1^ (irradiations at 21 °C, using Roza). The three doses achieved at this dose rate were equal to 67 kGy, 202 kGy, and 336 kGy.

To analyse gases emission and oxygen consumption, polymers have to be irradiated in closed containers. Hence, a second irradiation has to be performed, at doses as low as possible in comparison to the initial dose in order to maintain the material signature. The use of this two-step irradiation protocol has already been presented and has many advantages. For this second step of irradiation, polymers were placed in glass-closed ampoules, as presented in a previous article [21]. The atmosphere inside the containers was introduced at a known pressure before sealing. We chose reconstituted air (20.0% O_2_, 77.99% N_2_, and 2.01% Kr).

Two different irradiators were employed: a Gammacell^®^ (PCR, CEA Saclay, Gif-sur-Yvette, France), equipped with a 137Cs source and the POSEIDON facility (LABRA, CEA Saclay, Gif-sur-Yvette, France), equipped with a ^60^Co source. Gammacell^®^ was initially used to calibrate the mass to place in the glass ampoules: only one sample from each material was irradiated in such conditions because only 6 samples could be irradiated at the same time. Irradiation conditions were 0.29 kGy·h-1 (Fricke dosimeter) up to 22.3 kGy.

The POSEIDON facility was an industrial irradiator, so all of the sample of one pre-ageing condition (low, medium, and high dose rate from UJV irradiations) could be irradiated at the same time. Irradiation conditions were about 1 kGy·h^−1^ (UNIDOS PTW dosimeter equipped with a calibration chamber) up to 12 and 25 kGy (two glass ampoules were used for one sample, to validate the linearity of the concentration versus dose evolution). Exact irradiation conditions for all the samples can be found in Supplementary Information.

### 2.3. Gas Analyses and Radiation Chemical Yields Determination

Gas analyses were performed using a high-resolution gas mass spectrometer Thermo Fischer Scientific MAT-271 [21]. The instantaneous formation rate of gas X, $G_{D}\left(X\right)$, is determined using its partial pressure after irradiation in the closed container through Equation (1) given below.

(1) $$G_{D}\left(X\right)=\frac{1}{d}\cdot \frac{d\left[X\right]}{dt}=\frac{P_{f}\cdot {\%}_{vol,X}\cdot V_{free}}{R\cdot T\cdot \Delta D\cdot m}$$

where d is the dose rate in Gy·s^−1^, [*X*] is the concentration of the gas *X* in mol·kg^−1^ measured after irradiation at a given dose ΔD in Gy, $P_{f}$ is the total pressure in the glass ampoule at the end of the irradiation in Pa, ${\%}_{vol,X}$ is the gas X volume fraction, $V_{free}$ is the free volume in the glass ampoule in m^3^, *R* is the gas constant, *T* is the sample’s temperature under irradiation in K, and m is the mass of the irradiated sample in kg.

The uncertainty is always inferior to 10% in the hydrogen emission case; it is about 20% in the case of oxygen consumption and of carbon dioxide release.

### 2.4. Chemometric Analysis

#### 2.4.1. Principal Component Analysis (PCA)

Principal component analysis (PCA) is a tool commonly used in chemometrics and was described in a previous study [22]. Each principal component (PC) is built to maximise the variance extracted from the remaining data. The projection of the scores in the space defined by the PCs gives an overview of the similarities and differences between the samples, while the loadings indicate which variables bring more information to each PC [23]. In this study, we considered for each model only the PCs containing more than 15% of the variance. Thus, in some cases, only the first PC was relevant, and the scores could be represented as a bar chart. In other cases, the first two PCs were relevant, and the scores could be represented as a scatter plot.

#### 2.4.2. Anova in Common Dimensions (AComDim)

The Anova in Common Dimensions (AComDim) method allows highlighting of the influential factors (model γ-doses, dose rate, and modality) and their interactions by a simultaneous analysis of all data [15,24,25,26]. The AComDim is a multi-block analysis. The outputs of the AComDim method indicate whether variations in the data from different values relative to the change between two levels of a factor are significantly greater than the residual variability and, thus, meaningful. The AComDim method is based on the same concept as ANOVA-PCA (also called APCA), and its description can be found in the papers by Amat et al. [15] and Colombani et al. [16].

The AComDim method decomposes the experimental data matrix into successive matrices (also called mean matrices) containing the average at each level for each factor or interaction. The residuals matrix remaining after successive subtraction of all mean matrices is added back to each of them to obtain the means plus residuals matrices (called blocks). Then, a multi-block PCA of all matrices is performed in order to extract its first principal component, or the “Common Components” (CCs). Each block provides a specific contribution (i.e., a specific weight), called salience, to the definition of each common component. As all blocks contain a contribution from the residual matrix, the first Common Component CC1 (with higher saliences) contains mainly noise. The block significance is afterward estimated with a Fisher test (student Fisher *F*-test).

## 3. Results and Discussion

### 3.1. PCA Results

#### 3.1.1. Mod1

**Figure 1:**
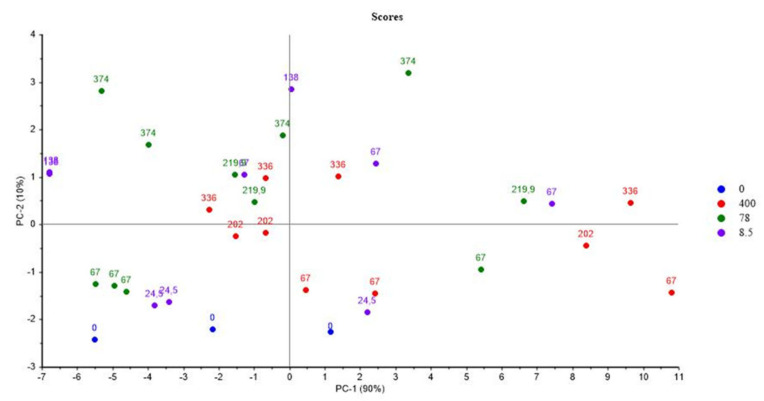
PCA results on Mod1: UJV irradiation dose rate (Gy/h) with doses (kGy) and irradiation locations.

**Figure 2:**
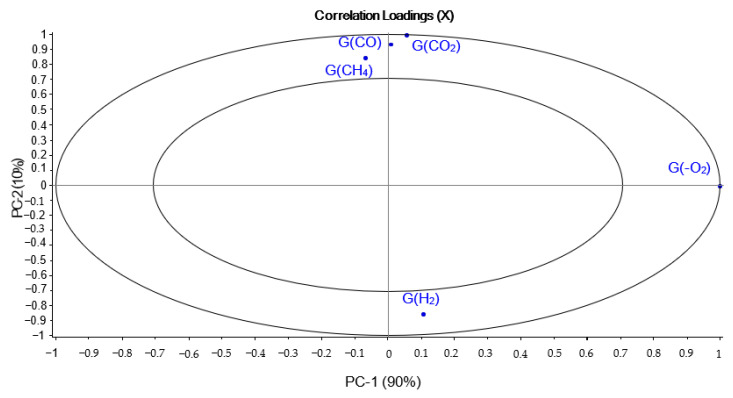
PCA loadings for PC1 and PC2 (Mod1).

Figure 1 and Figure 2 show the PCA scores and loadings for Mod1. The two first PC represent 100% of the total variance (90% for PC1 and 10% for PC2). We see that in Figure 1, the samples are dispersed, which shows a great inhomogeneity. The samples reirradiation location (PCR/LABRA) can be found on component 1, whereas on component 2, there is a rough distribution according to the initial irradiation dose. The evaluation of component 1 shows also that the oxidation is more important when the second step of irradiation is performed with the PCR Gammacell^®^ than when it is performed with the POSEIDON facility of the LABRA, this observation being true whatever the UJV irradiation dose.

Even at relatively low doses (67 kGy), the samples already have high levels of oxidation because they are found on the positive part of component 2. According to the loading (Figure 2), UJV low-dose samples present a high hydrogen radiation chemical yield G(H_2_), whereas UJV highest-dose samples present high G(CO), G(CO_2_), and G(CH_4_). These gases, and particularly carbon oxides (CO and CO_2_) release, are linked to the oxidation of the materials during irradiation: the higher the dose, the higher the degradation and the oxidation of the polymer [27]. This result is linked to the fact that this material has no added antioxidant in its composition, which means that it is not protected during radio-oxidation.

#### 3.1.2. Mod2

**Figure 3:**
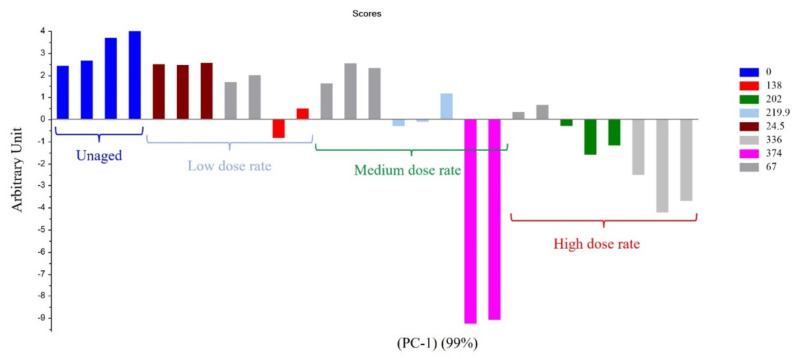
PC1 results on Mod2: first irradiation doses (kGy) classified as a function of dose rate.

**Figure 4:**
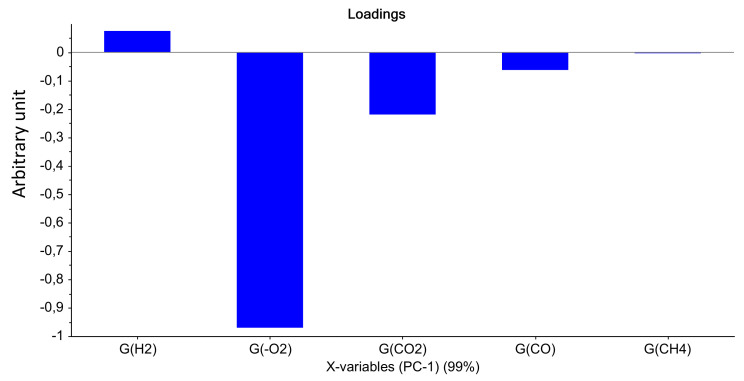
PCA loading for PC1 (Mod 2).

For Mod2, the first principal component explained 99% of the total variance. Figure 3 evidences that the most important parameter is the dose, this assertion being true whatever the dose rate. Only the highest UJV irradiation dose contributes to the difference (374 kGy for the 78 Gy·h^−1^ medium dose rate and more than 200 kGy for the 400 Gy·h^−1^ high dose rate). Moreover, according to Figure 4, low-UJV-irradiation-dose samples have a high G(H_2_), whereas high-UJV-irradiation-dose samples present important G(CO) and G(CO_2_), also indicating here the oxidation of the polymer at the most important doses of this study. Methane radiation chemical yield is not discriminant. Contrarily to the observation given in the case of Mod1, there is no impact of the PCR/LABRA second irradiation step on the PCA.

#### 3.1.3. Mod3

**Figure 5:**
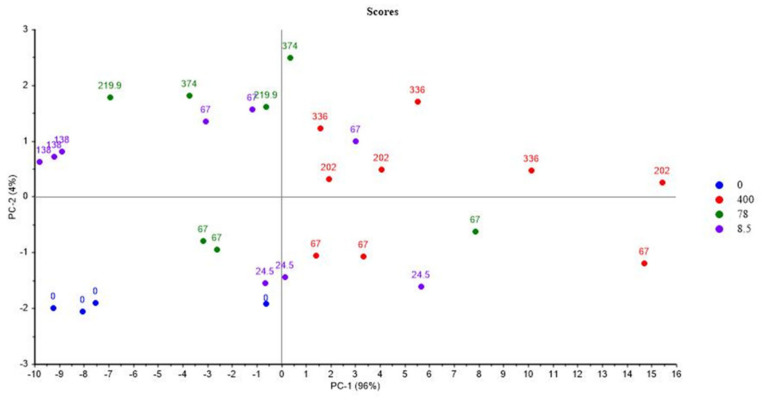
PCA results on Mod3: UJV irradiation dose rate (Gy/h) with location of the second step of irradiation.

**Figure 6:**
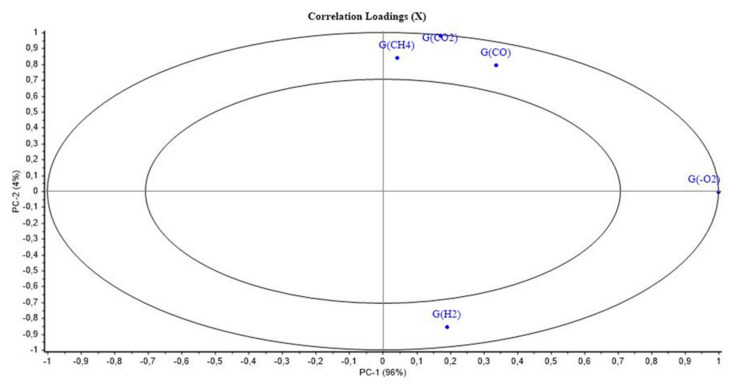
PCA loadings for PC1 and PC2 (Mod3).

Figure 5 and Figure 6 show the PCA scores and loadings for Mod3. The two first PCs represent 100% of the total variance (96% for PC1 and 4% for PC2). The first component is related to the location of the second step of irradiation that is PCR/LABRA. The second principal component is related to the UJV irradiation dose. Both results are very similar to those obtained in the case of Mod1.

According to Figure 6, UJV low-dose samples present a high G(H_2_), whereas UJV highest-dose samples present high G(CO), G(CO_2_), and G(CH_4_). G(-O_2_) is anti-correlated with these different gas releases. These results are linked to the oxidation of the materials during the UJV irradiation step. Figure 5 also shows that the oxidation is more important when the second step of irradiation is performed with the PCR Gammacell^®^ than when it is performed with the POSEIDON facility of the LABRA, this observation being true whatever the UJV irradiation dose.

As indicated just before, these results are very similar to those obtained for Mod1. The difference between both materials is that Irganox PS802 is added in the composition of Mod3. It might be deduced from these observations that this secondary antioxidant has no impact on the oxidation phenomenon in the conditions of this study.

#### 3.1.4. Mod4

**Figure 7:**
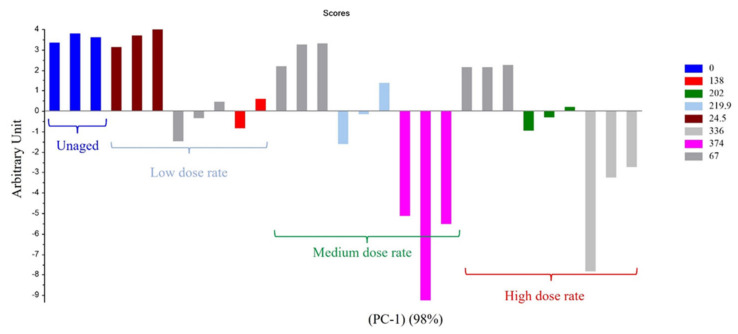
PC1 (98% of the variance) results on Mod4: first irradiation doses (kGy) with dose rates (Gy/h).

**Figure 8:**
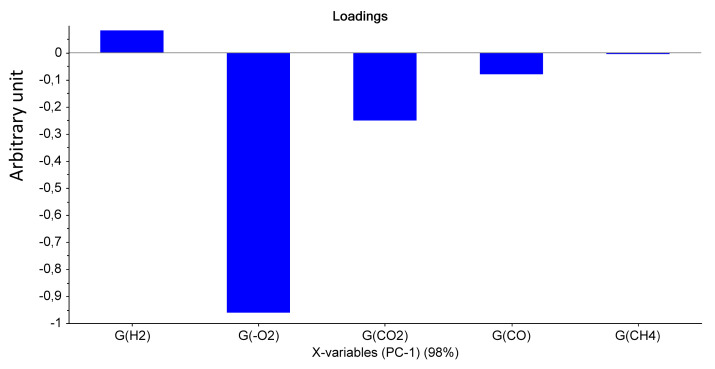
PCA loading for PC1 (Mod 4).

For Mod4, Figure 7 and Figure 8 evidence that the most important parameter is the dose, whatever the dose rate. Only the highest UJV irradiation dose contributes to the difference (374 kGy for the 78 Gy·h^−1^ medium dose rate and more than 200 kGy for the 400 Gy·h^−1^ high dose rate). There is no impact of the PCR/LABRA second irradiation step on the PCA.

Figure 7 shows that the first principal component (PC1) represents 98% of the total variance included in the data and corresponds to the UJV irradiation dose. Low-UJV-irradiation-dose samples have a high G(H_2_) and high-UJV-irradiation-dose samples present important G(CO) and G(CO_2_), also indicating here the oxidation of the polymer at the most important doses of this study. All these observations are very similar to observations given for Mod2.

#### 3.1.5. Mod5

**Figure 9:**
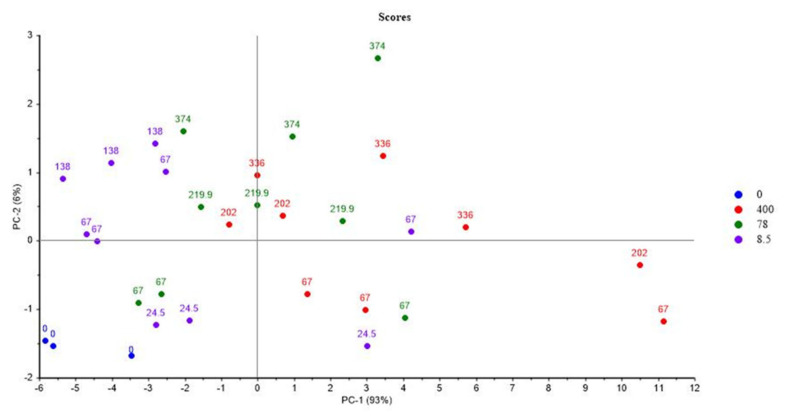
PCA results on Mod5: UJV irradiation dose rate (Gy/h) with location of the second step of irradiation.

**Figure 10:**
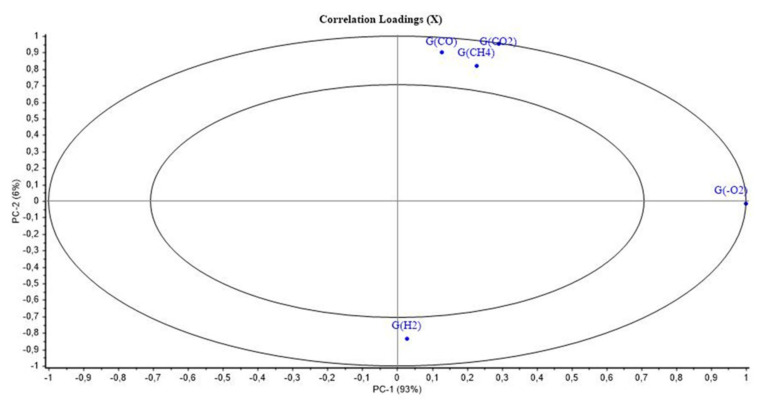
PCA loading for PC1 and PC2 (Mod 5).

Figure 9 and Figure 10 show the PCA scores and loadings for Mod5. The two first PCs represent 99% of the total variance (93% for PC1 and 6% for PC2). This first component is related to the location of the second step of irradiation that is PCR/LABRA, while the second principal component is related to the UJV irradiation dose and represents 6% of the total variance.

It is evidenced here that Mod5 has the same behaviour as those of Mod1 and Mod3. In fact, UJV low-dose samples present a high G(H_2_), whereas UJV highest-dose samples present high G(CO), G(CO_2_), and G(CH_4_), and G(-O_2_) is anti-correlated with these different gas releases. Oxidation is more important when the second step of irradiation is performed with the PCR Gammacell^®^ than when it is performed with the POSEIDON facility of the LABRA, this observation being true whatever the UJV irradiation dose.

All these results are similar to those obtained in the case of Mod1, evidencing that ATH has no influence on the aging of the material under irradiation.

#### 3.1.6. Mod6

**Figure 11:**
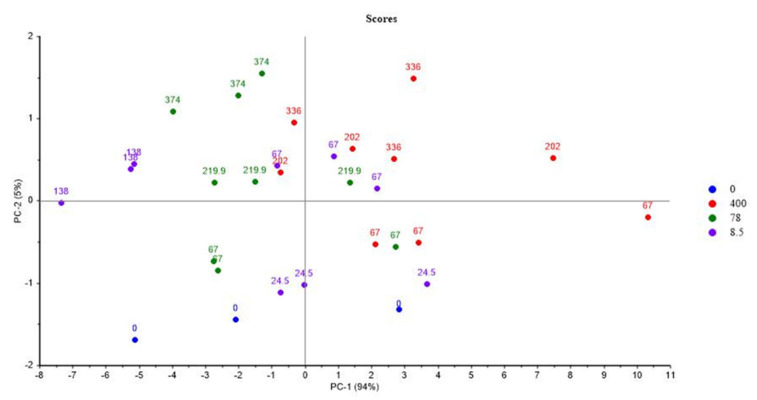
PCA results on Mod6: UJV irradiation dose rate (Gy/h) with location of the second step of irradiation.

**Figure 12:**
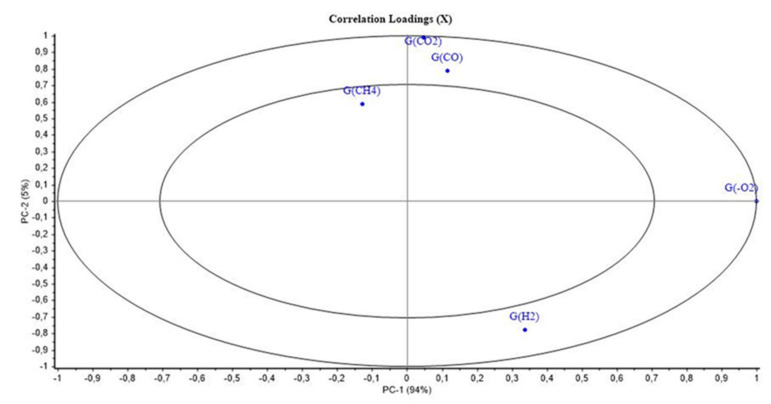
PCA loading for PC1 and PC2 (Mod 6).

Figure 11 and Figure 12 show the PCA scores and loadings for Mod6. The two first PC represent 99% of the total variance (94% for PC1 and 5% for PC2). The first principal component (PC1) is related to the location of the second step of irradiation (PCR/LABRA). The second principal component (PC2) is related to the UJV irradiation dose.

Figure 11 shows the PCA loadings for Mod6, which is similar to Mod1, Mod3, and Mod 5. In fact, UJV low-dose samples present a high G(H_2_), whereas UJV highest-dose samples present high G(CO) and G(CO_2_), and G(-O_2_) is anti-correlated with these different gas releases. Oxidation is more important when the second step of irradiation is performed with the PCR Gammacell^®^ than when it is performed with the POSEIDON facility of the LABRA, this observation being true whatever the UJV irradiation dose.

The only difference between Mod6 on one side and Mod1, Mod3, and Mod5 on the other side is that G(CH_4_) evolution is not discriminant for Mod6 (Figure 12).

#### 3.1.7. Mod7

**Figure 13:**
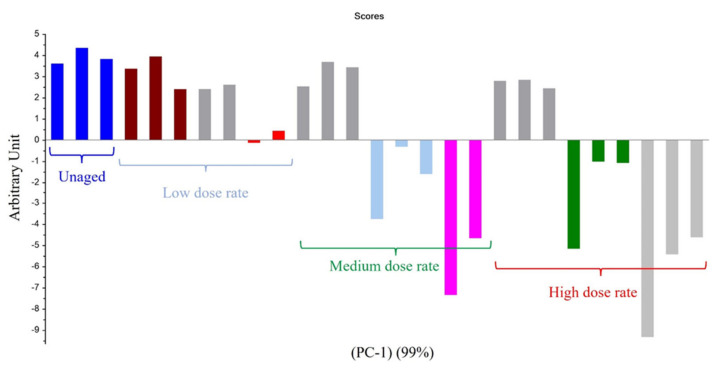
PC1 results on Mod7: first irradiation doses (kGy) with dose rates (Gy/h).

For Mod7, Figure 13 evidences that the most important parameter is the dose, whatever the dose rate. It represents 99% of the total variance, and only the highest UJV irradiation dose contributes to the difference (374 kGy for the 78 Gy·h^−1^ medium dose rate and more than 200 kGy for the 400 Gy·h^−1^ high dose rate). There is no impact of the PCR/LABRA second irradiation step on the PCA.

**Figure 14:**
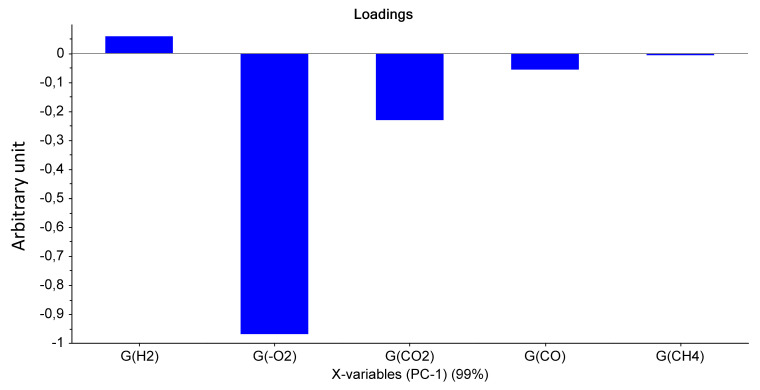
PCA loading for PC1 (Mod 7).

Figure 14 shows that low-UJV-irradiation-dose samples have a high G(H_2_) and high-UJV-irradiation-dose samples present important G(CO), G(CO_2_), and G(CH_4_), also indicating here the oxidation of the polymer at the most important doses of this study. All those observations are very similar to observations given for Mod2 and Mod4.

#### 3.1.8. All Models

**Figure 15:**
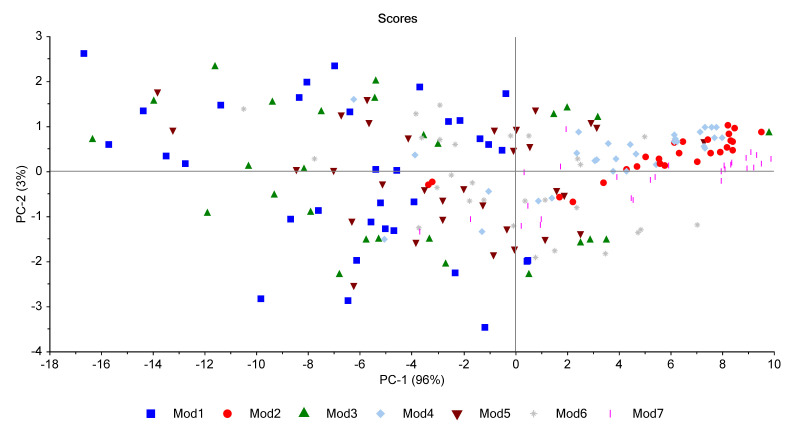
PCA results regrouping all models.

Figure 15 gathers results obtained for all the model materials (Mod1 to Mod7). It shows that the first principal component (PC1) represents 96% of the total variance and is linked to the irradiated material, which means to the additive added. In fact, Mod2, Mod4, and Mod7 seem to have the same behaviour and are gathered on the right side of PC1, while Mod1, Mod3, Mod5, and Mod6 are gathered on the left side of PC1. It should be reminded here that Mod2, Mod4, and Mod7 contain the Irganox 1076, whereas the four other model polymers do not contain this primary antioxidant.

**Figure 16:**
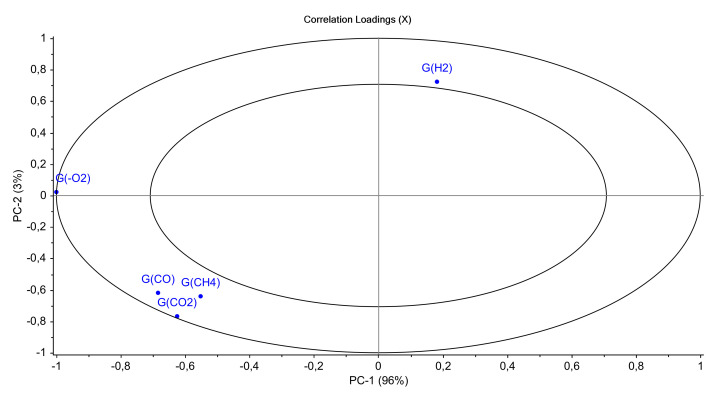
PCA loading for PC1 and PC2 (all models).

Figure 16 shows the PCA loadings for all materials: it is visible from this PCA that the most important effect is the presence of oxygen in the surrounding atmosphere of irradiation. Hydrogen release on one side, and carbon oxides and methane on the other side, are anti-correlated on the second component PCA-2. Nonetheless, this component represents only 3% of the variable analysis, which is negligible compared to PCA-1.

### 3.2. AComDim Results

As already indicated in the Introduction section, using AComDim gives highly interesting information about the variability in the quality indices resulting from the effect of polymer type, dose, dose rate, and modality of re-irradiation. The extracted Common Components (CCs), obtained with AComDim treatment, indicate if variations in the data between levels of a factor are significantly greater than the residual variability, that is if variations can be considered as relevant. Considering a data matrix of 208 re-irradiations, this multiblock method allowed the calculation of 15 common components. The number of common components is defined as the number of factors + their interactions + the noise (common component). In the present study, there are 4 factors (i.e., the polymer type, dose, dose rate, and process of re-irradiation), 10 interactions (1*2, 1*3, 1*4, 2*3, 3*4, 1*2*3, 1*2*4, 1*3*4, 2*3*4, and 1*2*3*4), and the noise, that is to say 4 + 10 components + the noise. The first Common Component CC1 corresponds to the effect of the residuals as all blocks contain a contribution from the residual error matrix, which was added back to each factor matrix. The less a block contributes to CC1, the more its source of variability is different from the residuals.

In this study, this AComDim method has evidenced that the most influential factors are the polymer type, the UJV irradiation dose, and, to a lesser extent, the interaction between these two parameters.

#### 3.2.1. Polymer Type Influence

**Figure 17:**
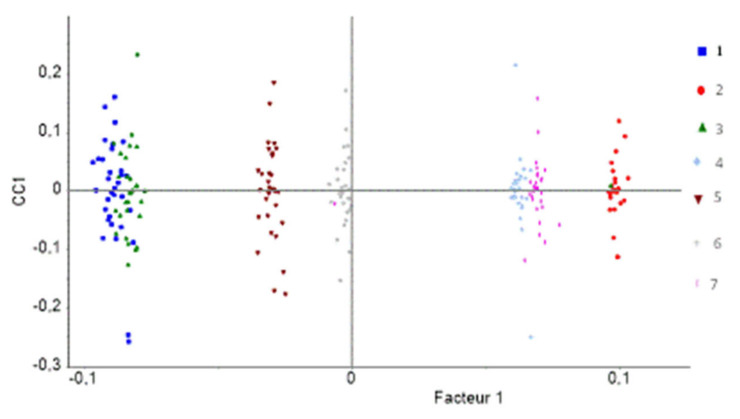
CC scores as a function of the polymers model type. In this analysis, 1: Mod1, 2: Mod2, 3: Mod3, 4: Mod4, 5: Mod5, 6: Mod6, and 7: Mod 7.

Figure 17 shows the AComDim scores obtained for the polymers model type. It can be observed that there is a discrimination between Mod1, Mod3, Mod5, and Mod6 which are on the left of Figure 17, and Mod2, Mod4, and Mod7 which lie on the right of Figure 17. This result confirms in another way all the results presented in the PCA results section: there are two categories of materials, those without primary antioxidant Irganox 1076 and those which contain this molecule.

It can moreover be observed that the most positive loadings correspond to Mod2 and reveal to be characteristic of the nondegraded polymer. The signature of the nondegraded polymer is a more important hydrogen release as compared to the exact same material but aged at different doses [15]. Polymers with a positive projection equivalent to Mod2 are Mod4 and Mod7: these samples also contain a polymer with little or no degradation. This implies that these materials are the less degraded ones and that even at the highest dose of this study, they are only degraded little. Surprisingly, the material containing only Irganox 1076 (Mod 2) seems less degraded that materials which contain Irganox PS802 (Mod 4) and Irganox PS802 plus ATH (Mod 7).

Mod5 and Mod6 are negatively projected, showing their deeper degradation under irradiation. Their degradation is not superimposed and is proportional to their organic content. In fact, Mod5 is more negatively loaded than Mod6.

It can also be observed that Mod1 and Mod3 on one side, and Mod4 and Mod7 on the other side, are superimposed. This observation allows confirmation of the conclusions given in the PCA results section, and in previous articles on the same materials [11,22]: there is absolutely no effect, under the conditions of this study, of the presence of the second antioxidant Irganox PS802 in the different XLPE.

It can be added that:

- Mod 1 behaves differently from Mod5 and Mod6;
- Mod7 behaves differently from Mod2 and Mod4.

**Figure 18:**
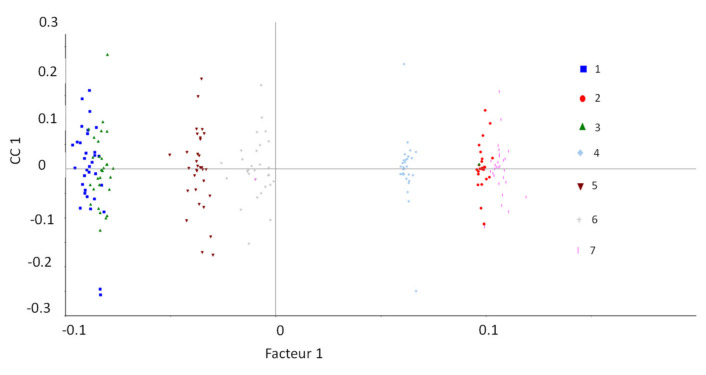
CC scores as a function of the polymers model type. Compared from Figure 17, radiation chemical yields have been normalised by the organic content in the polymer before being analysed. In this analysis, 1: Mod1, 2: Mod2, 3: Mod3, 4: Mod4, 5: Mod5, 6: Mod6, and 7: Mod 7.

These observations are at some point different from those obtained using PCAs (Figure 15). Nonetheless, by taking into account the quantity of organic matter in each material, that is, by dividing by $\left(100-ATH\;wt\%\right)$, Figure 17 can be redrawn as Figure 18 below.

Figure 18 evidences that Mod1, Mod3, Mod5, and Mod6 on one side and Mod2, Mod4, and Mod7 on the other side are closed. This shows in another way that the parameter of influence for these samples is the presence, or not, of Irganox 1076. Irganox PS802 and ATH have no effect at the chemical level on the gas emission and consumption.

**Figure 19:**
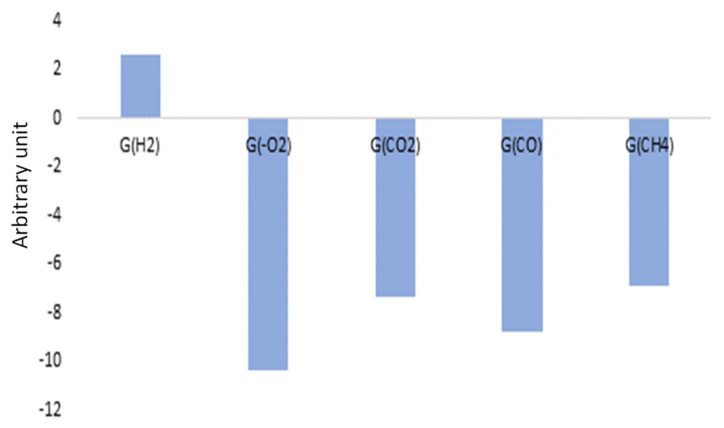
CCs loading depending on the polymer model type.

Finally, Figure 19 shows the associated loading depending on the gas emitted or consumed. The negative data on loading correspond to degraded polymers, i.e., those with high-oxygen-consumption radiation chemical yields, but also with important carbon oxides and methane emission radiation chemical yields. Positive data correspond to the nondegraded polymer. Hence, for Mod1, Mod3, Mod5, and Mod6, oxidation is the most influenced parameter, whereas for polymers containing Irganox 1076, hydrogen release is the most influencing factor.

#### 3.2.2. UJV Irradiation Dose Influence

**Figure 20:**
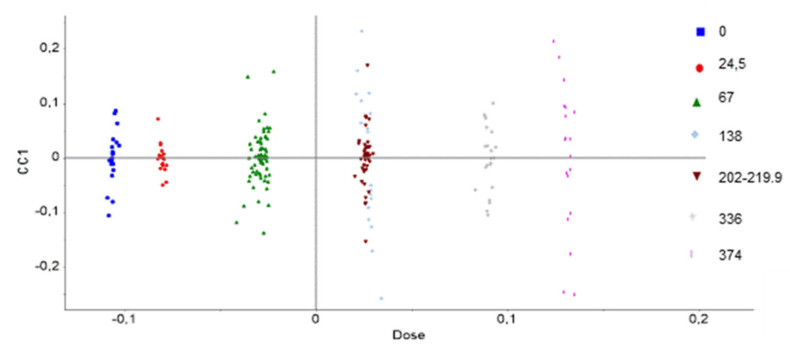
CC scores as a function of the UJV irradiation dose. In this analysis, the UJV irradiation doses are presented as follows: 1: 0 kGy, 2: 25 kGy (low dose rate), 3: 67 kGy (low, medium, and high dose rates), 4: 138 kGy (low dose rate), 5: 202 and 220 kGy (high and medium dose rates), 6: 336 kGy (high dose rate), 7: 374 kGy (medium dose rate).

Figure 20 shows the AComDim scores obtained for the UJV irradiation dose, the three dose rates altogether. It is obvious from this AComDim analysis that the higher the UJV irradiation dose, the higher the degradation.

An interesting point to observe on Figure 20 is the superposition of points 4 and 5, which means that there is no difference between an irradiation at 138 kGy at low dose rate and irradiations at 202 and 220 kGy at high and medium dose rates, respectively. This result is an example of the slight dose rate effect, which is introduced at the beginning of this AComDim results section.

It is known since the work of Decker and Mayo [27] that the higher the dose rate, the lower the degradation effect. The superposition of points 4 and 5 implies that the degradation at 138 kGy at a dose rate of 8.5 Gy·h^−1^ is similar to those obtained at more than 200 kGy at dose rates comprised between 78 and 400 Gy·h^−1^. It might be supposed that additives consumption will hence take place at a lower dose at low dose rates than at higher dose rates.

**Figure 21:**
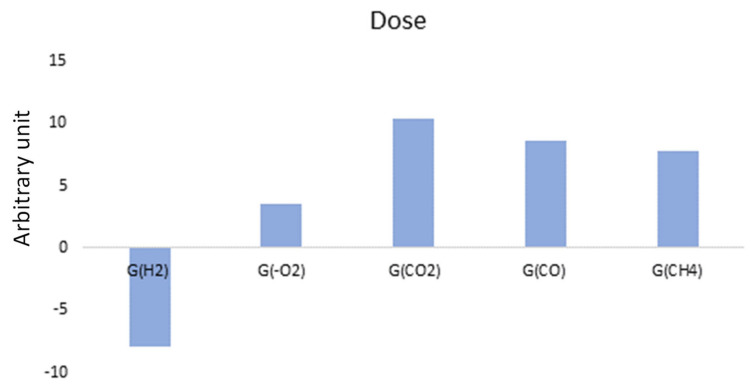
CCs loading depending on the UJV irradiation dose.

Associated loadings are presented in Figure 21. The low doses of this study are correlated to the negative loadings which correspond to hydrogen release radiation chemical yields: it implies that this gas is mostly released at low doses. On the contrary, at high doses, i.e., on the positive loadings, oxidation and associated gases become the most relevant influencing parameter. Numerical data and irradiation conditions can be found in Supplementary Materials.

## 4. Conclusions

With the effect of the addition of each additive separately using PCA, it has been shown that neither Irganox PS802 nor ATH are protecting at the molecular level the XLPE model polymer from ageing under the ageing conditions of this study. The only effect observed is the dilution effect due to the presence of inorganic ATH in an important weight amount. On the contrary, the presence of Irganox 1076 induces a protection against ageing that is very important, and which remains in the whole dose range domain of this study.

The presence or not of these additives induces changes in the radiation chemical yields. Each gas under study in this work is the signature of a degradation mechanism step. Hydrogen is mostly released when XLPE is only degraded little, whereas carbon oxides and methane are clearly linked to further degradation of oxidised materials. The nature of the gases emitted or consumed is linked to the dose effect, which has been identified as the second most important parameter of this study.

The effect of the location of the second step of irradiation (PCR versus LABRA) is linked to the well-known dose rate effect. This effect is far less highlighted in this study because of the additives’ presence: when a polymer is protected against ageing by efficient molecules, when Irganox 1076 is present in the case in this study, then the oxidation level of the material is low and starts to increase only when the protective molecules are fully consumed. In the materials under evaluation here, it is supposed that the primary antioxidant is fully consumed or almost fully only around the highest doses, i.e., 370 kGy. It would have been interesting to evaluate higher doses, but this was unfortunately not possible in the framework of this study.

The materials under study in the present article were evaluated using different analytical techniques and the obtained results have led to many articles, in addition to those already cited in this paper [28,29,30,31,32]. Some of the obtained results were analysed using PCA: it concerns FTIR spectra evaluation [22] and dielectric spectroscopy measurements [33]. The experimental results and their mathematical evaluation have led to equivalent conclusions. XLPE materials that are not protected by primary antioxidants are highly degraded at the highest doses of this study, whereas materials which contain Irganox 1076 are, on the contrary, relatively well protected. Moreover, Irganox PS802 and ATH are not protecting the different XLPE materials under the conditions of this study, when irradiations are performed at room temperature.

## Data Availability

Numerical data are given in Supplementary Materials.

## Supplementary Materials

The following supporting information can be downloaded at: https://www.mdpi.com/article/10.3390/polym14183810/s1. Numerical data and irradiation conditions can be found in Supplementary Information.Table S1: Radiation chemical yields, Table S2: Low dose rate, Table S3: Medium dose rate, Table S4: High dose rate.
