# Supplementary material for: Radio-Oxidation Ageing of XLPE Containing Different Additives and Filler: Principal Component Analyses of Gases Emission and Consumption
Source: Polymers (Basel). 2022 Sep 12;14(18):3810. doi: 10.3390/polym14183810 (PMC9503762; doi:10.3390/polym14183810)
Supplement: Supplementary file 1 [file polymers-14-03810-s001.zip › polymers-1852343-Supplementary Information.pdf]

Supplementary Information

Table S1. Radiation chemical yields.

	First-step irradiation		Second-step irradiation			Radiation chemical yields (10^{-7} mol.J^{-1})				
Database ID	Dose D (kGy)	Dose rate *d'* (kGy/h)	Irradiator	Dose ΔD (kGy)	Dose rate *d* (kGy/h)	G(H_2)	G(-O_2)	G(CO_2)	G(CO)	G(CH_4)
Mod1-Tpe-UnA-Msgas-CEA-a	0	-	PCR	22.3	0.29	3.73	21.3	0.99	0.67	0.034
Mod1-Tpe-UnA-Msgas-CEA-b	0	-	LABRA	12.0	1.01	3.72	14.6	0.75	0.53	0.052
Mod1-Tpe-UnA-Msgas-CEA-c	0	-	LABRA	24.0	1.01	3.63	17.9	0.98	0.64	0.014
Mod2-Tpe-UnA-Msgas-CEA-a	0	-	PCR	22.3	0.29	3.57	6.0	0.29	0.20	0.016
Mod2-Tpe-UnA-Msgas-CEA-b	0	-	PCR	22.3	0.29	3.50	5.8	0.29	0.24	0.004
Mod2-Tpe-UnA-Msgas-CEA-c	0	-	LABRA	12.0	1.01	3.46	4.7	0.22	0.18	0.021
Mod2-Tpe-UnA-Msgas-CEA-d	0	-	LABRA	24.0	1.01	3.40	4.4	0.19	0.15	0.019
Mod3-Tpe-UnA-Msgas-CEA-a	0	-	PCR	22.3	0.29	3.41	19.6	0.96	0.78	0.034
Mod3-Tpe-UnA-Msgas-CEA-b	0	-	LABRA	12.0	1.01	3.55	12.2	0.69	0.52	0.043
Mod3-Tpe-UnA-Msgas-CEA-c	0	-	LABRA	12.7	1.06	3.18	11.0	0.59	0.42	0.029
Mod3-Tpe-UnA-Msgas-CEA-d	0	-	LABRA	25.44	1.06	3.09	12.7	0.71	0.48	0.031
Mod4-Tpe-UnA-Msgas-CEA-a	0	-	PCR	22.3	0.29	3.40	6.9	0.36	0.32	0.022
Mod4-Tpe-UnA-Msgas-CEA-b	0	-	LABRA	12.0	1.01	3.32	6.4	0.28	0.29	0.022
Mod4-Tpe-UnA-Msgas-CEA-c	0	-	LABRA	24.0	1.01	3.31	6.6	0.29	0.29	0.026
Mod5-Tpe-UnA-Msgas-CEA-a	0	-	PCR	22.3	0.29	3.11	13.4	0.68	0.56	0.038
Mod5-Tpe-UnA-Msgas-CEA-b	0	-	LABRA	24.0	1.01	3.01	11.3	0.65	0.49	0.049
Mod5-Tpe-UnA-Msgas-CEA-c	0	-	LABRA	12.7	1.06	2.71	6.9	0.39	0.39	0.032
Mod5-Tpe-UnA-Msgas-CEA-d	0	-	LABRA	25.44	1.06	2.69	11.1	0.60	0.45	0.031
Mod6-Tpe-UnA-Msgas-CEA-a	0	-	PCR	22.3	0.29	2.67	17.1	0.88	0.60	0.040
Mod6-Tpe-UnA-Msgas-CEA-b	0	-	LABRA	12.0	1.01	2.65	9.2	0.55	0.45	0.050
Mod6-Tpe-UnA-Msgas-CEA-c	0	-	LABRA	24.0	1.01	2.66	12.2	0.78	0.53	0.048
Mod7-Tpe-UnA-Msgas-CEA-a	0	-	PCR	22.3	0.29	2.37	5.1	0.25	0.20	0.024
Mod7-Tpe-UnA-Msgas-CEA-b	0	-	LABRA	12.0	1.01	2.33	4.3	0.28	0.26	0.029
Mod7-Tpe-UnA-Msgas-CEA-c	0	-	LABRA	24.0	1.01	2.39	4.9	0.29	0.24	0.031

Table S2. Low dose rate

Database ID	First-step irradiation		Second-step irradiation			Radiation chemical yields (10^{-7} mol.J^{-1})				
	Dose D (kGy)	Dose rate *d'* (kGy/h)	Irradiator	Dose ΔD (kGy)	Dose rate *d* (kGy/h)	G(H_2)	G(-O_2)	G(CO_2)	G(CO)	G(CH_4)
Mod1-Tpe-RA(RT)5-25-Msgas-CEA-a	24.5	5	PCR	20.0	0.29	2.83	22.3	1.11	0.65	0.026
Mod1-Tpe-RA(RT)5-25-Msgas-CEA-b	24.5	5	LABRA	12.0	0.99	2.67	16.7	1.23	0.65	0.046
Mod1-Tpe-RA(RT)5-25-Msgas-CEA-c	24.5	5	LABRA	24.0	0.99	2.57	16.3	1.06	0.80	0.024
Mod2-Tpe-RA(RT)5-25-Msgas-CEA-a	24.5	5	PCR	20.0	0.29	3.18	5.9	0.32	0.17	0.013
Mod2-Tpe-RA(RT)5-25-Msgas-CEA-b	24.5	5	LABRA	12.0	0.99	3.12	5.9	0.43	0.29	0.015
Mod2-Tpe-RA(RT)5-25-Msgas-CEA-c	24.5	5	LABRA	24.0	0.99	3.03	5.8	0.41	0.25	0.018
Mod3-Tpe-RA(RT)5-25-Msgas-CEA-a	24.5	5	PCR	20.0	0.29	3.12	25.9	1.37	0.73	0.038
Mod3-Tpe-RA(RT)5-25-Msgas-CEA-b	24.5	5	LABRA	12.0	0.99	3.16	19.6	1.23	0.92	0.032
Mod3-Tpe-RA(RT)5-25-Msgas-CEA-c	24.5	5	LABRA	24.0	0.99	3.07	20.4	1.32	1.05	0.034
Mod4-Tpe-RA(RT)5-25-Msgas-CEA-a	24.5	5	PCR	20.0	0.29	3.18	7.1	0.42	0.22	0.019
Mod4-Tpe-RA(RT)5-25-Msgas-CEA-b	24.5	5	LABRA	12.0	0.99	3.08	6.5	0.41	0.29	0.015
Mod4-Tpe-RA(RT)5-25-Msgas-CEA-c	24.5	5	LABRA	24.0	0.99	2.97	6.2	0.34	0.30	0.015
Mod5-Tpe-RA(RT)5-25-Msgas-CEA-a	24.5	5	PCR	20.0	0.29	2.38	19.9	1.04	0.42	0.024
Mod5-Tpe-RA(RT)5-25-Msgas-CEA-b	24.5	5	LABRA	12.0	0.99	2.31	15.0	1.04	0.53	0.025
Mod5-Tpe-RA(RT)5-25-Msgas-CEA-c	24.5	5	LABRA	24.0	0.99	2.32	14.1	0.86	0.75	0.019
Mod6-Tpe-RA(RT)5-25-Msgas-CEA-a	24.5	5	PCR	20.0	0.29	1.99	18.0	0.94	0.38	0.025
Mod6-Tpe-RA(RT)5-25-Msgas-CEA-b	24.5	5	LABRA	12.0	0.99	2.02	14.3	0.94	0.54	0.021
Mod6-Tpe-RA(RT)5-25-Msgas-CEA-c	24.5	5	LABRA	24.0	0.99	1.96	13.6	0.80	0.63	0.019
Mod7-Tpe-RA(RT)5-25-Msgas-CEA-a	24.5	5	PCR	20.0	0.29	2.25	5.3	0.35	0.34	0.016
Mod7-Tpe-RA(RT)5-25-Msgas-CEA-c	24.5	5	LABRA	24.0	0.99	2.06	4.7	0.34	0.24	0.014
Mod1-Tpe-RA(RT)5-67-Msgas-CEA-a	67.1	5	PCR	18.8	0.28	2.07	27.5	3.32	0.93	0.085
Mod1-Tpe-RA(RT)5-67-Msgas-CEA-b	67.1	5	LABRA	12.0	0.99	1.95	22.5	4.08	1.19	0.093
Mod1-Tpe-RA(RT)5-67-Msgas-CEA-c	67.1	5	LABRA	24.0	0.99	1.93	18.8	3.77	1.24	0.109
Mod2-Tpe-RA(RT)5-67-Msgas-CEA-b	67.1	5	LABRA	12.0	0.99	2.93	6.6	0.75	0.37	0.022
Mod2-Tpe-RA(RT)5-67-Msgas-CEA-c	67.1	5	LABRA	24.0	0.99	2.82	6.3	0.61	0.37	0.000

Database ID	First-step irradiation		Second-step irradiation			Radiation chemical yields (10^{-7} mol.J^{-1})				
	Dose D (kGy)	Dose rate *d'* (kGy/h)	Irradiator	Dose ΔD (kGy)	Dose rate *d* (kGy/h)	$G(H_2)$	$G(-O_2)$	$G(CO_2)$	G(CO)	$G(CH_4)$
Mod3-Tpe-RA(RT)5-67-Msgas-CEA-a	67.1	5	PCR	18.8	0.28	2.24	23.2	3.67	1.37	0.078
Mod3-Tpe-RA(RT)5-67-Msgas-CEA-b	67.1	5	LABRA	12.0	0.99	2.07	19.0	4.09	1.43	0.078
Mod3-Tpe-RA(RT)5-67-Msgas-CEA-c	67.1	5	LABRA	24.0	0.99	2.03	17.1	3.80	1.39	0.090
Mod4-Tpe-RA(RT)5-67-Msgas-CEA-a	67.1	5	PCR	18.8	0.28	3.29	11.7	1.09	0.47	0.034
Mod4-Tpe-RA(RT)5-67-Msgas-CEA-b	67.1	5	LABRA	12.0	0.99	3.03	10.5	1.09	0.52	0.030
Mod4-Tpe-RA(RT)5-67-Msgas-CEA-c	67.1	5	LABRA	24.0	0.99	2.92	9.7	0.92	0.56	0.023
Mod5-Tpe-RA(RT)5-67-Msgas-CEA-a	67.1	5	PCR	18.8	0.28	1.74	21.0	2.61	0.85	0.068
Mod5-Tpe-RA(RT)5-67-Msgas-CEA-c	67.1	5	LABRA	24.0	0.99	1.57	12.4	1.78	0.79	0.030
Mod5-Tpe-RA(RT)5-67-Msgas-CEA-b	67.1	5	LABRA	24.0	1.06	1.59	12.1	1.90	0.70	0.054
Mod5-Tpe-RA(RT)5-67-Msgas-CEA-c	67.1	5	LABRA	24.0	1.06	1.56	14.2	2.98	0.97	0.057
Mod6-Tpe-RA(RT)5-67-Msgas-CEA-a	67.1	5	PCR	18.8	0.28	1.44	16.5	1.94	0.69	0.051
Mod6-Tpe-RA(RT)5-67-Msgas-CEA-b	67.1	5	LABRA	12.0	0.99	1.38	15.2	2.35	0.70	0.052
Mod6-Tpe-RA(RT)5-67-Msgas-CEA-c	67.1	5	LABRA	24.0	0.99	1.34	13.5	2.22	0.75	0.058
Mod7-Tpe-RA(RT)5-67-Msgas-CEA-a	67.1	5	LABRA	12.0	0.99	1.97	6.2	0.70	0.31	0.021
Mod7-Tpe-RA(RT)5-67-Msgas-CEA-b	67.1	5	LABRA	24.0	0.99	1.89	6.2	0.67	0.26	0.020
Mod7-Tpe-RA(RT)5-67-Msgas-CEA-b	67.1	5	LABRA	24.0	1.06	1.93	6.0	0.61	0.31	0.018
Mod1-Tpe-RA(RT)5-138-Msgas-CEA-a	138	5	LABRA	12.0	0.99	1.66	20.1	5.57	1.60	0.111
Mod1-Tpe-RA(RT)5-138-Msgas-CEA-a	138	5	LABRA	24.0	1.06	1.62	13.3	3.59	1.28	0.082
Mod1-Tpe-RA(RT)5-138-Msgas-CEA-b	138	5	LABRA	24.0	1.06	1.59	13.3	3.60	1.36	0.087
Mod2-Tpe-RA(RT)5-138-Msgas-CEA-a	138	5	LABRA	12.0	0.99	2.89	9.1	1.13	0.54	0.029
Mod2-Tpe-RA(RT)5-138-Msgas-CEA-b	138	5	LABRA	24.0	0.99	2.85	7.8	0.86	0.41	0.000
Mod3-Tpe-RA(RT)5-138-Msgas-CEA-b	138	5	LABRA	24.0	0.99	1.66	10.4	2.69	1.20	0.038
Mod3-Tpe-RA(RT)5-138-Msgas-CEA-b	138	5	LABRA	24.0	1.06	1.66	11.0	2.85	0.99	0.058
Mod3-Tpe-RA(RT)5-138-Msgas-CEA-b	138	5	LABRA	24.0	1.06	1.63	11.3	2.95	0.97	0.063
Mod4-Tpe-RA(RT)5-138-Msgas-CEA-a	138	5	LABRA	12.0	0.99	2.72	10.9	1.35	0.79	0.034
Mod4-Tpe-RA(RT)5-138-Msgas-CEA-b	138	5	LABRA	24.0	0.99	2.79	9.5	1.04	0.65	0.023

	First-step irradiation		Second-step irradiation			Radiation chemical yields (10^{-7} mol.J^{-1})				
**Database ID**	**Dose D (kGy)**	**Dose rate *d'* (kGy/h)**	**Irradiator**	**Dose ΔD (kGy)**	**Dose rate *d* (kGy/h)**	**G(H_2)**	**G(-O_2)**	**G(CO_2)**	**G(CO)**	**G(CH_4)**
Mod5-Tpe-RA(RT)5-138-Msgas-CEA-b	138	5	LABRA	24.0	0.99	1.34	11.4	2.54	1.12	0.042
Mod5-Tpe-RA(RT)5-138-Msgas-CEA-b	138	5	LABRA	24.0	1.06	1.40	13.9	3.32	1.11	0.069
Mod5-Tpe-RA(RT)5-138-Msgas-CEA-b	138	5	LABRA	24.0	1.06	1.36	12.7	2.93	0.98	0.038
Mod6-Tpe-RA(RT)5-138-Msgas-CEA-b	138	5	LABRA	24.0	0.99	1.11	7.0	1.65	0.78	0.029
Mod6-Tpe-RA(RT)5-138-Msgas-CEA-b	138	5	LABRA	24.00	1.06	1.13	9.1	2.12	0.68	0.051
Mod6-Tpe-RA(RT)5-138-Msgas-CEA-b	138	5	LABRA	24.00	1.06	1.13	9.2	2.18	0.72	0.048
Mod7-Tpe-RA(RT)5-138-Msgas-CEA-a	138	5	LABRA	12.0	0.99	1.89	8.7	1.12	0.46	0.027
Mod7-Tpe-RA(RT)5-138-Msgas-CEA-b	138	5	LABRA	24.0	0.99	1.84	8.2	0.77	0.40	0.014

Table S3. Medium dose rate

	First-step irradiation		Second-step irradiation			Radiation chemical yields (10^{-7} mol.J^{-1})				
**Database ID**	**Dose D (kGy)**	**Dose rate *d'* (kGy/h)**	**Irradiator**	**Dose ΔD (kGy)**	**Dose rate *d* (kGy/h)**	**G(H_2)**	**G(-O_2)**	**G(CO_2)**	**G(CO)**	**G(CH_4)**
Mod1-Tpe-RA(RT)40-67-Msgas-CEA-a	67.1	40	PCR	20.0	0.29	2.45	25.5	1.96	0.75	0.045
Mod1-Tpe-RA(RT)40-67-Msgas-CEA-b	67.1	40	LABRA	12.7	1.06	2.30	15.5	1.33	0.55	0.037
Mod1-Tpe-RA(RT)40-67-Msgas-CEA-c	67.1	40	LABRA	12.0	1.07	2.61	15.1	1.57	0.57	0.040
Mod1-Tpe-RA(RT)40-67-Msgas-CEA-d	67.1	40	LABRA	24.0	1.07	2.43	14.6	1.55	0.56	0.034
Mod2-Tpe-RA(RT)40-67-Msgas-CEA-a	67.1	40	PCR	20.0	0.29	3.15	6.7	0.55	0.27	0.044
Mod2-Tpe-RA(RT)40-67-Msgas-CEA-b	67.1	40	LABRA	12.7	1.06	2.75	5.8	0.47	0.29	0.010
Mod2-Tpe-RA(RT)40-67-Msgas-CEA-c	67.1	40	LABRA	25.4	1.06	2.79	6.0	0.48	0.21	0.019
Mod3-Tpe-RA(RT)40-67-Msgas-CEA-a	67.1	40	PCR	20.0	0.29	2.43	28.1	2.23	0.81	0.060
Mod3-Tpe-RA(RT)40-67-Msgas-CEA-b	67.1	40	LABRA	12.7	1.06	2.41	17.7	1.58	0.69	0.047
Mod3-Tpe-RA(RT)40-67-Msgas-CEA-c	67.1	40	LABRA	25.4	1.06	2.50	17.1	1.76	0.71	0.063
Mod4-Tpe-RA(RT)40-67-Msgas-CEA-a	67.1	40	PCR	20.0	0.29	3.12	8.0	0.67	0.35	0.026
Mod4-Tpe-RA(RT)40-67-Msgas-CEA-b	67.1	40	LABRA	12.7	1.06	2.77	6.9	0.56	0.31	0.024
Mod4-Tpe-RA(RT)40-67-Msgas-CEA-c	67.1	40	LABRA	25.4	1.06	2.71	6.8	0.56	0.28	0.023

Mod5-Tpe-RA(RT)40-67-Msgas-CEA-a	67.1	40	PCR	20.0	0.29	2.17	20.9	1.43	0.63	0.036
Mod5-Tpe-RA(RT)40-67-Msgas-CEA-b	67.1	40	LABRA	12.7	1.06	1.98	13.6	1.12	0.42	0.028
Mod5-Tpe-RA(RT)40-67-Msgas-CEA-c	67.1	40	LABRA	25.4	1.06	2.02	14.2	1.31	0.47	0.036
Mod6-Tpe-RA(RT)40-67-Msgas-CEA-a	67.1	40	PCR	20.0	0.29	1.62	17.1	1.26	0.53	0.000
Mod6-Tpe-RA(RT)40-67-Msgas-CEA-b	67.1	40	LABRA	12.7	1.06	1.61	11.7	1.02	0.33	0.025
Mod6-Tpe-RA(RT)40-67-Msgas-CEA-c	67.1	40	LABRA	25.4	1.06	1.63	11.6	1.13	0.37	0.027
Mod7-Tpe-RA(RT)40-67-Msgas-CEA-a	67.1	40	PCR	20.0	0.29	2.18	6.1	0.64	0.34	0.030
Mod7-Tpe-RA(RT)40-67-Msgas-CEA-b	67.1	40	LABRA	12.7	1.06	1.92	4.9	0.42	0.21	0.015
Mod7-Tpe-RA(RT)40-67-Msgas-CEA-c	67.1	40	LABRA	25.4	1.06	1.91	5.2	0.47	0.21	0.018

	**First-step irradiation**		**Second-step irradiation**			**Radiation chemical yields (10^{-7} mol.J^{-1})**				
**Database ID**	**Dose D (kGy)**	**Dose rate *d'* (kGy/h)**	**Irradiator**	**Dose ΔD (kGy)**	**Dose rate *d* (kGy/h)**	**G(H_2)**	**G(-O_2)**	**G(CO_2)**	**G(CO)**	**G(CH_4)**
Mod1-Tpe-RA(RT)40-220-Msgas-CEA-a	219.9	40	PCR	20.0	0.29	1.97	26.7	3.31	1.03	0.075
Mod1-Tpe-RA(RT)40-220-Msgas-CEA-b	219.9	40	LABRA	12.7	1.06	1.87	19.1	3.17	1.03	0.079
Mod1-Tpe-RA(RT)40-220-Msgas-CEA-d	219.9	40	LABRA	24.0	1.07	1.80	18.5	3.74	1.11	0.087
Mod2-Tpe-RA(RT)40-220-Msgas-CEA-a	219.9	40	PCR	20.0	0.29	2.85	8.6	1.12	0.39	0.012
Mod2-Tpe-RA(RT)40-220-Msgas-CEA-b	219.9	40	LABRA	12.7	1.06	2.58	8.3	1.12	0.45	0.032
Mod2-Tpe-RA(RT)40-220-Msgas-CEA-c	219.9	40	LABRA	25.4	1.06	2.97	7.1	1.05	0.36	0.035
Mod3-Tpe-RA(RT)40-220-Msgas-CEA-a	219.9	40	LABRA	12.7	1.06	1.85	19.5	4.07	1.44	0.108
Mod3-Tpe-RA(RT)40-220-Msgas-CEA-b	219.9	40	LABRA	25.4	1.06	2.05	13.2	4.27	0.75	0.126
Mod4-Tpe-RA(RT)40-220-Msgas-CEA-a	219.9	40	PCR	20.0	0.29	2.87	11.7	1.42	0.57	0.038
Mod4-Tpe-RA(RT)40-220-Msgas-CEA-b	219.9	40	LABRA	12.7	1.06	2.61	10.2	1.21	0.56	0.034
Mod4-Tpe-RA(RT)40-220-Msgas-CEA-c	219.9	40	LABRA	25.4	1.06	2.97	8.6	1.34	0.46	0.039
Mod5-Tpe-RA(RT)40-220-Msgas-CEA-a	219.9	40	PCR	20.0	0.29	1.53	19.1	2.57	0.80	0.062
Mod5-Tpe-RA(RT)40-220-Msgas-CEA-b	219.9	40	LABRA	12.7	1.06	1.52	16.8	2.65	0.79	0.063
Mod5-Tpe-RA(RT)40-220-Msgas-CEA-c	219.9	40	LABRA	25.4	1.06	1.37	15.2	2.50	0.57	0.055
Mod6-Tpe-RA(RT)40-220-Msgas-CEA-a	219.9	40	PCR	20.0	0.29	1.38	15.7	2.01	0.61	0.060
Mod6-Tpe-RA(RT)40-220-Msgas-CEA-b	219.9	40	LABRA	12.7	1.06	1.26	12.8	2.00	0.55	0.074

Mod6-Tpe-RA(RT)40-220-Msgas-CEA-c	219.9	40	LABRA	25.4	1.06	1.24	11.6	2.00	0.55	0.049
Mod7-Tpe-RA(RT)40-220-Msgas-CEA-a	219.9	40	PCR	20.0	0.29	1.97	12.3	1.40	0.57	0.031
Mod7-Tpe-RA(RT)40-220-Msgas-CEA-b	219.9	40	LABRA	12.7	1.06	1.76	8.9	1.16	0.48	0.029
Mod7-Tpe-RA(RT)40-220-Msgas-CEA-c	219.9	40	LABRA	25.4	1.06	1.72	10.2	1.26	0.47	0.028
Mod1-Tpe-RA(RT)40-374-Msgas-CEA-a	374	40	PCR	20.0	0.29	1.46	19.9	4.43	1.55	0.090
Mod1-Tpe-RA(RT)40-374-Msgas-CEA-b	374	40	LABRA	12.7	1.06	1.75	14.7	5.48	1.70	0.125
Mod1-Tpe-RA(RT)40-374-Msgas-CEA-c	374	40	LABRA	25.4	1.06	1.36	16.1	4.22	1.16	0.092
Mod1-Tpe-RA(RT)40-374-Msgas-CEA-d	374	40	LABRA	12.0	1.07	1.57	23.4	5.92	1.71	0.056
Mod2-Tpe-RA(RT)40-374-Msgas-CEA-a	374	40	LABRA	12.7	1.06	2.36	17.3	2.72	0.92	0.016
Mod2-Tpe-RA(RT)40-374-Msgas-CEA-b	374	40	LABRA	25.4	1.06	2.19	17.2	2.55	0.83	0.056

	**First-step irradiation**		**Second-step irradiation**			**Radiation chemical yields (10^{-7} mol.J^{-1})**				
**Database ID**	**Dose D (kGy)**	**Dose rate *d'* (kGy/h)**	**Irradiator**	**Dose ΔD (kGy)**	**Dose rate *d* (kGy/h)**	**G(H_2)**	**G(-O_2)**	**G(CO_2)**	**G(CO)**	**G(CH_4)**
Mod3-Tpe-RA(RT)40-374-Msgas-CEA-a	374	40	LABRA	12.7	1.06	1.48	20.5	4.91	1.46	0.106
Mod3-Tpe-RA(RT)40-374-Msgas-CEA-b	374	40	LABRA	25.4	1.06	1.46	16.4	4.10	1.19	0.095
Mod4-Tpe-RA(RT)40-374-Msgas-CEA-c	374	40	LABRA	25.4	1.06	2.21	15.0	2.47	0.94	0.053
Mod4-Tpe-RA(RT)40-374-Msgas-CEA-d	374	40	LABRA	12.0	1.07	2.06	18.8	4.07	1.31	0.098
Mod4-Tpe-RA(RT)40-374-Msgas-CEA-e	374	40	LABRA	24.0	1.07	1.97	15.2	3.35	1.11	0.070
Mod5-Tpe-RA(RT)40-374-Msgas-CEA-a	374	40	PCR	20.0	0.29	1.39	17.7	3.68	1.24	0.076
Mod5-Tpe-RA(RT)40-374-Msgas-CEA-b	374	40	LABRA	25.4	1.06	1.22	14.6	3.52	1.02	0.082
Mod5-Tpe-RA(RT)40-374-Msgas-CEA-c	374	40	LABRA	12.0	1.07	1.32	19.9	5.01	1.47	0.104
Mod6-Tpe-RA(RT)40-374-Msgas-CEA-a	374	40	PCR	20.0	0.29	1.08	12.3	3.06	0.74	0.062
Mod6-Tpe-RA(RT)40-374-Msgas-CEA-b	374	40	LABRA	12.7	1.06	0.99	13.0	3.26	0.97	0.068
Mod6-Tpe-RA(RT)40-374-Msgas-CEA-c	374	40	LABRA	25.4	1.06	1.00	10.4	2.82	0.77	0.064
Mod7-Tpe-RA(RT)40-374-Msgas-CEA-a	374	40	LABRA	12.7	1.06	1.27	15.7	2.84	0.84	0.059
Mod7-Tpe-RA(RT)40-374-Msgas-CEA-b	374	40	LABRA	25.4	1.06	1.24	12.9	2.58	0.74	0.058

Table S4. High dose rate

	First-step irradiation		Second-step irradiation			Radiation chemical yields (10^{-7} mol.J^{-1})				
**Database ID**	**Dose D (kGy)**	**Dose rate *d'* (kGy/h)**	**Irradiator**	**Dose ΔD (kGy)**	**Dose rate *d* (kGy/h)**	**$G(H_2)$**	**$G(-O_2)$**	**$G(CO_2)$**	**G(CO)**	**$G(CH_4)$**
Mod1-Tpe-RA(RT)300-67-Msgas-CEA-a	67	300	PCR	20.0	0.29	3.05	30.9	1.72	0.80	0.041
Mod1-Tpe-RA(RT)300-67-Msgas-CEA-b	67	300	LABRA	12.0	1.07	2.86	22.5	1.53	0.77	0.042
Mod1-Tpe-RA(RT)300-67-Msgas-CEA-c	67	300	LABRA	24.0	1.07	2.74	20.6	1.57	0.68	0.062
Mod2-Tpe-RA(RT)300-67-Msgas-CEA-a	67	300	LABRA	12.0	1.07	2.92	8.0	0.69	0.28	0.027
Mod2-Tpe-RA(RT)300-67-Msgas-CEA-b	67	300	LABRA	24.0	1.07	2.86	7.7	0.59	0.27	0.021
Mod3-Tpe-RA(RT)300-67-Msgas-CEA-a	67	300	PCR	20.0	0.29	2.98	34.9	1.99	1.01	0.046
Mod3-Tpe-RA(RT)300-67-Msgas-CEA-b	67	300	LABRA	12.0	1.07	2.83	23.6	1.73	0.94	0.067
Mod3-Tpe-RA(RT)300-67-Msgas-CEA-c	67	300	LABRA	24.0	1.07	2.74	21.6	1.67	0.91	0.055
Mod4-Tpe-RA(RT)300-67-Msgas-CEA-a	67	300	PCR	20.0	0.29	3.15	8.1	0.61	0.31	0.022
Mod4-Tpe-RA(RT)300-67-Msgas-CEA-b	67	300	LABRA	12.0	1.07	2.89	8.0	0.63	0.38	0.026
Mod4-Tpe-RA(RT)300-67-Msgas-CEA-c	67	300	LABRA	24.0	1.07	2.85	7.9	0.62	0.34	0.024
Mod5-Tpe-RA(RT)300-67-Msgas-CEA-a	67	300	PCR	20.0	0.29	2.36	28.0	1.96	0.68	0.037
Mod5-Tpe-RA(RT)300-67-Msgas-CEA-b	67	300	LABRA	24.0	1.07	2.31	19.8	1.59	0.41	0.040
Mod5-Tpe-RA(RT)300-67-Msgas-CEA-c	67	300	LABRA	12.0	0.99	2.28	18.2	1.65	0.71	0.031
Mod6-Tpe-RA(RT)300-67-Msgas-CEA-a	67	300	PCR	20.0	0.29	2.01	24.7	1.76	0.63	0.035
Mod6-Tpe-RA(RT)300-67-Msgas-CEA-b	67	300	LABRA	12.0	1.07	1.94	17.7	1.44	0.63	0.039
Mod6-Tpe-RA(RT)300-67-Msgas-CEA-c	67	300	LABRA	24.0	1.07	1.88	16.5	1.39	0.65	0.035
Mod7-Tpe-RA(RT)300-67-Msgas-CEA-a	67	300	PCR	20.0	0.29	2.19	5.8	0.58	0.27	0.020
Mod7-Tpe-RA(RT)300-67-Msgas-CEA-b	67	300	LABRA	12.0	1.07	3.60	6.9	0.55	0.49	0.030
Mod7-Tpe-RA(RT)300-67-Msgas-CEA-c	67	300	LABRA	24.0	1.07	2.26	5.8	0.57	0.25	0.004
Mod7-Tpe-RA(RT)300-67-Msgas-CEA-d	67	300	LABRA	12.0	0.99	2.02	6.1	0.83	0.82	0.025
Mod1-Tpe-RA(RT)300-202-Msgas-CEA-a	202	300	PCR	20.0	0.29	2.51	28.5	2.49	1.06	0.058
Mod1-Tpe-RA(RT)300-202-Msgas-CEA-b	202	300	LABRA	12.0	1.07	2.88	19.4	2.81	1.12	0.068
Mod1-Tpe-RA(RT)300-202-Msgas-CEA-c	202	300	LABRA	24.0	1.07	2.77	18.6	2.70	1.04	0.068
	**First-step irradiation**		**Second-step irradiation**			**Radiation chemical yields (10^{-7} mol.J^{-1})**				

	First-step irradiation		Second-step irradiation			Radiation chemical yields (10^{-7} mol.J^{-1})				
**Database ID**	**Dose D (kGy)**	**Dose rate *d'* (kGy/h)**	**Irradiator**	**Dose ΔD (kGy)**	**Dose rate *d* (kGy/h)**	**$G(H_2)$**	**$G(-O_2)$**	**$G(CO_2)$**	**G(CO)**	**$G(CH_4)$**
Mod2-Tpe-RA(RT)300-202-Msgas-CEA-a	202	300	PCR	20.0	0.29	2.84	8.5	1.23	0.45	0.014
Mod2-Tpe-RA(RT)300-202-Msgas-CEA-b	202	300	LABRA	12.0	1.07	3.08	9.8	1.64	0.53	0.045
Mod2-Tpe-RA(RT)300-202-Msgas-CEA-c	202	300	LABRA	24.0	1.07	2.97	9.4	1.47	0.48	0.046
Mod3-Tpe-RA(RT)300-202-Msgas-CEA-a	202	300	PCR	20.0	0.29	2.44	35.6	3.36	1.11	0.063
Mod3-Tpe-RA(RT)300-202-Msgas-CEA-b	202	300	LABRA	12.0	1.07	2.84	24.2	3.39	1.32	0.095
Mod3-Tpe-RA(RT)300-202-Msgas-CEA-c	202	300	LABRA	24.0	1.07	2.74	22.1	3.13	1.25	0.078
Mod4-Tpe-RA(RT)300-202-Msgas-CEA-a	202	300	PCR	20.0	0.29	2.68	11.0	1.40	0.59	0.036
Mod4-Tpe-RA(RT)300-202-Msgas-CEA-b	202	300	LABRA	12.0	1.07	3.01	10.3	1.71	0.67	0.050
Mod4-Tpe-RA(RT)300-202-Msgas-CEA-c	202	300	LABRA	24.0	1.07	2.93	9.8	1.59	0.65	0.041
Mod5-Tpe-RA(RT)300-202-Msgas-CEA-a	202	300	PCR	20.0	0.29	2.03	27.3	2.65	0.81	0.051
Mod5-Tpe-RA(RT)300-202-Msgas-CEA-b	202	300	LABRA	12.0	1.07	2.26	17.4	2.81	0.91	0.071
Mod5-Tpe-RA(RT)300-202-Msgas-CEA-c	202	300	LABRA	24.0	1.07	2.16	16.0	2.54	0.83	0.057
Mod6-Tpe-RA(RT)300-202-Msgas-CEA-a	202	300	PCR	20.0	0.29	1.73	21.8	2.43	0.79	0.045
Mod6-Tpe-RA(RT)300-202-Msgas-CEA-b	202	300	LABRA	12.0	1.07	1.78	15.7	2.59	0.95	0.050
Mod6-Tpe-RA(RT)300-202-Msgas-CEA-c	202	300	LABRA	24.0	1.07	1.58	13.6	2.21	0.85	0.008
Mod7-Tpe-RA(RT)300-202-Msgas-CEA-a	202	300	PCR	20.0	0.29	1.82	13.7	1.68	0.58	0.039
Mod7-Tpe-RA(RT)300-202-Msgas-CEA-b	202	300	LABRA	12.0	1.07	1.86	9.4	1.79	0.58	0.043
Mod7-Tpe-RA(RT)300-202-Msgas-CEA-c	202	300	LABRA	24.0	1.07	1.74	9.5	1.68	0.61	0.041
Mod1-Tpe-RA(RT)300-336-Msgas-CEA-a	336	300	PCR	20.0	0.29	2.30	29.7	3.42	1.09	0.070
Mod1-Tpe-RA(RT)300-336-Msgas-CEA-b	336	300	LABRA	12.0	1.07	2.60	21.4	3.99	1.34	0.082
Mod1-Tpe-RA(RT)300-336-Msgas-CEA-b	336	300	LABRA	24.0	1.06	2.15	19.4	3.71	1.52	0.074
Mod1-Tpe-RA(RT)300-336-Msgas-CEA-c	336	300	LABRA	24.0	1.06	2.11	17.8	3.00	1.32	0.075
Mod2-Tpe-RA(RT)300-336-Msgas-CEA-a	336	300	PCR	20.0	0.29	2.61	10.6	1.87	0.58	0.046
Mod2-Tpe-RA(RT)300-336-Msgas-CEA-b	336	300	LABRA	12.0	1.07	2.84	12.2	2.55	0.75	0.064
Mod2-Tpe-RA(RT)300-336-Msgas-CEA-c	336	300	LABRA	24.0	0.99	2.45	11.7	2.35	0.98	0.056

Database ID	Dose D (kGy)	Dose rate *d'* (kGy/h)	Irradiator	Dose ΔD (kGy)	Dose rate *d* (kGy/h)	$G(H_2)$	$G(-O_2)$	$G(CO_2)$	G(CO)	$G(CH_4)$
Mod3-Tpe-RA(RT)300-336-Msgas-CEA-a	336	300	PCR	20.0	0.29	2.25	30.3	3.33	1.26	0.040
Mod3-Tpe-RA(RT)300-336-Msgas-CEA-b	336	300	LABRA	12.0	1.07	2.52	25.7	4.60	1.52	0.101
Mod3-Tpe-RA(RT)300-336-Msgas-CEA-c	336	300	LABRA	24.0	1.07	2.28	21.7	3.91	1.36	0.091
Mod4-Tpe-RA(RT)300-336-Msgas-CEA-a	336	300	PCR	20.0	0.29	2.42	17.9	2.07	0.99	0.057
Mod4-Tpe-RA(RT)300-336-Msgas-CEA-b	336	300	LABRA	12.0	1.07	2.59	13.0	2.58	1.04	0.060
Mod4-Tpe-RA(RT)300-336-Msgas-CEA-c	336	300	LABRA	24.0	1.07	2.39	12.6	2.34	1.03	0.059
Mod5-Tpe-RA(RT)300-336-Msgas-CEA-a	336	300	PCR	20.0	0.29	1.81	22.5	2.80	0.93	0.066
Mod5-Tpe-RA(RT)300-336-Msgas-CEA-b	336	300	LABRA	12.0	1.07	2.05	20.1	3.85	1.08	0.085
Mod5-Tpe-RA(RT)300-336-Msgas-CEA-c	336	300	LABRA	24.0	1.07	1.84	16.7	3.21	1.02	0.075
Mod6-Tpe-RA(RT)300-336-Msgas-CEA-a	336	300	PCR	20.0	0.29	1.51	17.0	2.35	0.83	0.044
Mod6-Tpe-RA(RT)300-336-Msgas-CEA-b	336	300	LABRA	12.0	1.07	1.68	17.6	3.44	1.18	0.069
Mod6-Tpe-RA(RT)300-336-Msgas-CEA-c	336	300	LABRA	24.0	1.07	1.54	14.0	2.85	0.97	0.061
Mod7-Tpe-RA(RT)300-336-Msgas-CEA-a	336	300	PCR	20.0	0.29	1.58	17.5	3.53	1.07	0.178
Mod7-Tpe-RA(RT)300-336-Msgas-CEA-b	336	300	LABRA	12.0	1.07	1.76	13.7	2.94	0.90	0.068
Mod7-Tpe-RA(RT)300-336-Msgas-CEA-c	336	300	LABRA	24.0	1.07	1.59	12.9	2.60	0.80	0.052
